# Innovations in biomarker stratification for precision oncology

**DOI:** 10.1007/s10238-026-02150-2

**Published:** 2026-04-28

**Authors:** Ronak Naeemaee, Keith Harris, Neil Cross, Jon Griffin, Lewis A. Quayle

**Affiliations:** 1 School of Computing and Digital Technologies, Sheffield Hallam University, Sheffield, UK; 2 School of Biosciences and Chemistry, Sheffield Hallam University, Sheffield, UK; 3 Division of Clinical Medicine, School of Medicine and Population Health, University of Sheffield, Sheffield, UK; 4 Department of Histopathology, Sheffield Teaching Hospitals NHS Foundation Trust, Sheffield, UK

**Keywords:** Predictive biomarkers, Cancer prognosis, Prognostic modelling, Machine learning, Data-driven methods, Clinical validation

## Abstract

Biomarker stratification underpins precision oncology, yet survival analysis often relies on arbitrary thresholds that undermine reproducibility and clinical relevance, particularly for continuous biomarkers. This review focuses on methodological approaches for stratifying continuous biomarkers within survival analysis frameworks, examining conventional strategies alongside data-driven and machine learning methods in the context of threshold selection and clinical interpretability. We evaluate the extent to which these approaches address key challenges including heterogeneity, confounding, and overfitting, and critically appraise their strengths and limitations for clinically actionable risk stratification. By synthesising current evidence, we highlight opportunities for more robust and reproducible prognostic modelling and outline future directions to improve the reliability of biomarker-driven decision-making in oncology.

## Introduction

Precision oncology is built on the principle that treatments should be tailored to the biological and clinical characteristics of individual patients. Central to this approach is the identification of reliable biomarkers that can stratify patients into clinically meaningful groups, enabling improved outcome prediction and more effective treatment selection. In oncology, survival analysis provides the statistical foundation for evaluating how biomarkers relate to outcomes such as overall survival, disease-specific survival, progression-free survival, or distant metastasis–free survival [1]. By accounting for censored data and modelling risk over time, survival analysis has become a cornerstone of biomarker research [2]. The Kaplan-Meier estimator is widely used to compare survival between categorical groups (e.g., high versus low expression/abundance), while the Cox proportional hazards model can incorporate continuous covariates directly - though both approaches often hinge on careful threshold selection for continuous biomarkers.

Despite its central role in precision oncology, survival analysis faces well-recognised challenges when applied to biomarker stratification. Traditional methods are well suited to discrete variables such as tumour stage or mutation status, where natural categories exist. However, an increasing number of biomarkers - particularly those generated by high-throughput technologies such as next-generation sequencing or mass spectrometry - are continuous in nature. Unlike discrete biomarkers, continuous variables lack obvious thresholds for defining clinically relevant subgroups. To overcome this, researchers often rely on arbitrary cut points, such as splitting a cohort at the median value or dividing it into tertiles or quartiles. Although simple and easy to apply, these strategies risk oversimplifying complex biological relationships and ultimately limiting clinical translation [3, 4].

The use of arbitrary cut points has several important limitations. These include obscuring non-linear associations between biomarker levels and outcomes, reducing statistical power, and increasing the risk of false-positive or false-negative findings. Arbitrary stratification also fails to account for the biological and clinical heterogeneity that characterises most cancers [5, 6]. For example, intratumoural heterogeneity means that bulk assays tend to average across spatially and temporally distinct subclones and may miss the most aggressive component driving prognosis and treatment response [7]. This averaging effect is likely to be a major confounding factor underlying some of the apparent non-linear biomarker-outcome relationships observed in survival analyses [8]. In addition, differences in tumour biology, patient demographics, and treatment regimens can all influence biomarker-outcome relationships, yet these factors are rarely incorporated when fixed thresholds are applied in routine practice.

There are, however, examples of good practice in which thresholds are derived in large, well-annotated cohorts and subsequently prespecified and tested in independent validation datasets. Notable examples include the development and validation of multigene assays such as Oncotype DX and MammaPrint in breast cancer [9, 10]. Nevertheless, such rigorous strategies remain the exception rather than the rule in biomarker-driven survival analyses. As a result, biomarkers that appear promising in discovery studies frequently fail to validate in independent cohorts, hindering their clinical translation [11, 12]. This methodological weakness also has more immediate clinical implications: inaccurate cut points may lead to misleading risk stratification, inappropriate treatment decisions, and patients either being denied potentially beneficial therapies or exposed to unnecessary toxicities [13]. For precision oncology to deliver on its promise, more robust approaches to biomarker stratification are urgently needed - approaches that capture the complexity of biological data while remaining interpretable and clinically actionable.

In this review, we summarise the current landscape of biomarker stratification, focussing on the challenge of stratifying continuous biomarkers within survival analysis frameworks, while highlighting both strengths and limitations of existing methods. We begin by contrasting discrete and continuous biomarkers and outlining how arbitrary stratification is typically applied. We then discuss standard survival analysis frameworks, such as the Kaplan-Meier estimator and the Cox proportional hazards model, alongside their inherent limitations when applied to continuous data. Building on this foundation, we review advances in data-driven cut point identification, statistical modelling, and machine learning, with particular emphasis on threshold selection, interpretability, and their clinical relevance. We also explore emerging innovations that integrate molecular and clinical covariates, address overfitting and reproducibility, and strengthen validation practices. Finally, we provide a high-level conceptual framework for the assessment of biomarkers in precision oncology. Throughout, our aim is to offer a balanced and clinically oriented perspective on how methodological advances can enhance biomarker stratification and, ultimately, improve patient outcomes in oncology.

## Conventional Strategies for biomarker stratification: limitations in practice

Biomarkers in oncology span a spectrum from discrete (nominal and ordinal) to inherently continuous types. Discrete biomarkers - such as EGFR mutations in non-small cell lung cancer (NSCLC) or KRAS mutations in colorectal cancer (both nominal), as well as tumour stage, histological grade, or TMA-derived cell counts (ordinal) - lend themselves well to survival analysis because they provide naturally categorical inputs. However, many such variables originate from underlying continuous measurements that have been categorised, sometimes arbitrarily. For example, tumour stage can mask variation in invasion depth (e.g., pT1 bladder cancers ranging from minimal epithelial invasion to near-muscle invasive), Breslow thickness in melanoma is frequently binned, and HER2 IHC scoring collapses continuous expression into + 2/+3 categories [4, 13]. Even nominally discrete mutations can carry continuous prognostic information through variant allele fraction and molecular subtype, yet these are often simplified for clinical decision-making [14]. By contrast, inherently continuous biomarkers - such as gene or protein expression levels, tumour mutational burden, or immune infiltration scores - exist on a true spectrum without natural thresholds [4, 13]. These variables offer the potential for more nuanced prognostic and predictive information, but they also present the core challenge addressed throughout this review: defining clinically meaningful thresholds that enable patient group comparisons, survival analysis, and practical translation.

**Fig. 1:**
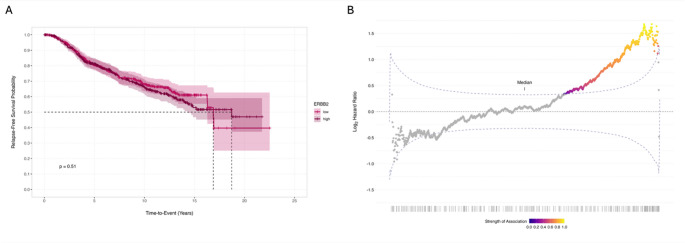
Median split failure and data-driven ERBB2 stratification in METABRIC. (**A**) Kaplan-Meier curves for relapse-free survival using a median ERBB2 expression split in the METABRIC discovery cohort (*n* = 995) show no significant separation between “high” and “low” expression groups (log-rank *p* = 0.51), illustrating a false-negative result when an arbitrary threshold is applied. (**B**) Exhaustive survival analysis of all possible dichotomisation points across the ordered ERBB2 expression range demonstrates that prognostic effects vary continuously, with 344 cut points showing a statistically significant association with outcome, none of which lie at or near the median. For each potential cut point, log_2_ hazard ratios are estimated and compared against a bootstrap-derived null distribution (10,000 random permutations) to obtain confidence limits and significance, and a “strength of association” score combines scaled hazard ratios and p-values so that only cut points with both large effect size and strong statistical support are highlighted

The most common solution has been to impose arbitrary cut points on continuous data, dividing patients into “high” and “low” groups based on the median, tertiles, quartiles, or other percentiles of biomarker expression [15]. Alternatively, thresholds may be borrowed from prior clinical studies or expert consensus, even when they lack statistical validation in the dataset at hand [16]. These approaches are attractive because they are simple, quick to apply, and easy for clinicians to interpret. A median split, for example, ensures equal group sizes and produces results that are readily visualised using Kaplan-Meier survival curves. However, these benefits come at a cost. Arbitrary cut points implicitly assume a step-like relationship, ignore the possibility of gradual or non-linear effects, and discard much of the information contained in continuous data. Perhaps most importantly, arbitrary stratification does not account for cohort heterogeneity. Patient age, comorbidities, treatment regimens, and tumour characteristics can all influence the biomarker-outcome relationship. When these factors are ignored, thresholds identified in one study often fail to replicate in another, limiting their clinical utility [15, 17]. Figure 1 provides an illustrative demonstration of this issue: median ERBB2 splits in the METABRIC discovery cohort (*n* = 995) yield a false-negative result (log-rank *p* = 0.51), whereas exhaustive analysis identifies 344 potential cut points far from the median that are all significantly associated with survival outcomes. While this example is intended for illustration rather than generalisation, it is particularly instructive given that ERBB2 encodes HER2 - the therapeutic target of trastuzumab (Herceptin) - and that METABRIC represents one of the largest and most clinically representative open-access breast cancer datasets.

Compared to categorical approaches, the Cox proportional hazards model offers more flexibility, as it can incorporate continuous predictors directly and estimate their effect on hazard over time. This model has become a mainstay of survival analysis because it accommodates censored data and provides interpretable hazard ratios [18]. Nonetheless, the average log-linear effect per unit increase (or per standard deviation) across the biomarker range resulting from continuous covariate incorporation, though excellent for inference, does not provide a directly actionable threshold for clinical decision making. Moreover, it is built on key assumptions that frequently fail in practice. Chief among these is the proportional hazards assumption - that hazard ratios remain constant over time. This can be tested using log-log plots or by assessing Schoenfeld residuals, but it is nonetheless violated in many cancer biomarker studies, particularly in high-dimensional or heterogeneous datasets where biomarker effects may vary over time or across patient subgroups [19]. While the model is reasonably robust to moderate violations, hazards in this setting can diverge substantially over time [20–22]. One notable example of this being that early benefit from HER2-targeted therapy may not persist in the long-term [23]. In addition, the model assumes a linear relationship between continuous biomarker values and the log hazard, which rarely reflects biological reality and may obscure non-linear or threshold effects that are clinically meaningful [19]. To address some of these limitations, more complex model extensions have been developed, including ridge, lasso, or elastic net penalisation for high-dimensional data and time-varying coefficients to relax proportional hazards. However, these refinements can introduce additional complexity without resolving the core issue: the need to identify robust, clinically meaningful thresholds for continuous biomarkers [24]. Alternative frameworks, including flexible parametric survival models, multi-state models, and competing risks approaches, may address some of these limitations in specific contexts, although their application to biomarker thresholding remains less clearly defined [25, 26].

Across both arbitrary stratification and standard survival analysis approaches, several limitations persist. First, arbitrary cut points increase the risk of both false-positive and false-negative results, particularly in small or underpowered studies where random variation dominates [4, 13]. A threshold may appear statistically significant in one dataset purely by chance, leading to spurious associations. Conversely, biologically meaningful patterns may be missed if they do not align with the chosen cut point [27]. Second, heterogeneity within patient cohorts complicates biomarker analysis. Differences in tumour biology, demographic variables, and treatment exposures can shift the apparent prognostic value of a biomarker. As a result, a cut point that is valid in one context may not apply in another, undermining reproducibility [5, 6]. Finally, many survival models remain vulnerable to oversimplification: di- or polychotomising a continuous biomarker reduces data richness and masks non-linear effects, while models treating biomarkers as continuous often rely on restrictive assumptions that fail across patient populations [27, 28].

Collectively, these challenges help to explain why fewer than 1% of published cancer biomarkers reach clinical practice [11]. They also highlight the need for more sophisticated, data-driven methods that capture cancer’s biological complexity while preserving interpretability and reproducibility for robust clinical decision-making.

## Data-driven and machine learning approaches for biomarker stratification

To overcome the limitations of arbitrary thresholds, several statistical methods have been developed to identify cut points directly from the data. These “data-driven” approaches aim to optimise the separation between patient groups while preserving the prognostic information contained in continuous biomarkers.

One widely used technique is the minimum p-value approach, which systematically tests potential cut points and selects the one yielding the strongest association with outcome [29–31]. Although simple, this strategy inflates false-positive risk through multiple testing and can lead to unstable thresholds and misclassification. For example, gene expression signatures optimised via minimising p-value in diffuse large B-cell lymphoma showed strong prognostic promise in discovery cohorts but failed external validation [32]. Statistical corrections including permutation-based adjustment, Bonferroni correction, and false discovery rate control have been proposed to mitigate these effects, though they do not fully resolve issues of instability and reproducibility, and the method remains sensitive to chance fluctuations [33]. A related family of methods involves maximally selected statistics, which identify thresholds that maximise survival separation [33, 34]. These provide a more formal framework than median or quantile splits and may better align with biological effects, but they can remain unstable in small or heterogeneous datasets [6, 33]. Receiver operating characteristic (ROC) curve-based methods, such as Youden’s Index and time-dependent ROC approaches, offer further alternatives that can handle censoring and balance sensitivity with specificity. These methods are widely used because they provide clinically interpretable measures of diagnostic performance, though they too can be influenced by cohort composition and outcome prevalence [35–38]. Collectively, these approaches offer more principled threshold selection than arbitrary cut points, but they remain vulnerable to overfitting. As with any threshold-search strategy, rigorous multi-step validation is essential, including internal cross-validation, external cohort testing, platform-independent reproducibility (e.g., microarrays versus RNA-seq), and feasibility of clinical implementation (i.e., lab-standardised cut points) [11, 39, 40].

Beyond cut point identification, some statistical models incorporate continuous biomarkers directly into survival analysis. The Cox proportional hazards model with threshold search extends the traditional framework by testing multiple candidate thresholds within the model itself. This allows formal evaluation of cut points while retaining hazard ratio interpretability. However, the approach remains limited by reliance on proportional hazards and can be unstable in the presence of correlated biomarkers [24]. More flexible alternatives include generalised additive models (GAMs), which relax linearity assumptions by modelling biomarker effects as smooth curves. GAMs are particularly useful for non-linear or non-monotonic (e.g., U-shaped) relationships - patterns observed in substantial fraction (around 20–30%) of biomarker analyses, including immunotherapy response variation across PD-L1 expression levels [41]. However, GAMs can be computationally intensive and their practical implementation requires careful selection of smoothing parameters, as over-smoothing may obscure clinically relevant structure while under-smoothing risks overfitting, particularly in small datasets. These approaches shift the emphasis away from artificial categorisation and towards capturing the biomarker’s functional relationship with outcome, though translating smooth functional relationships into clinically actionable decision rules and balancing flexibility with clinical interpretability remain key challenges [42].

Machine learning (ML) methods offer powerful alternatives, particularly for high-dimensional multi-omics datasets. Unlike conventional models, ML approaches can capture complex non-linear biomarker-outcome interactions without assuming linearity or proportional hazards [43, 44]. Unsupervised methods (e.g., clustering, principal component analysis, and latent class analysis) are valuable for identifying novel prognostic subtypes at the cohort-level; increasingly, single-sample gene expression classifiers derived from such approaches are entering evaluation for personalised medicine [45]. However, resulting subtype clusters are not always clinically interpretable, and robust validation for deployment remains essential [46, 47]. Supervised tree-based models, such as random survival forests and optimal survival trees, are popular in oncology because they balance predictive accuracy with interpretability [48, 49]. Support vector machines and deep learning approaches can achieve superior predictive performance, particularly when integrating genomic and imaging data, but they face “black-box” transparency barriers and require careful validation to ensure robustness across heterogeneous datasets, which can limit adoption in routine medical practice. In response to these limitations, explainable AI methods (e.g., Local Interpretable Model-agnostic Explanations or LIME and SHapley Additive exPlanations or SHAP) are increasingly incorporated into biomarker research to support transparency and clinical trust, although practical challenges such as class imbalance, batch effects, and missing data may still influence model performance in applied settings [50, 51].

Table 1 provides a comparative overview of the major methods discussed in this section, highlighting their strengths, limitations, and clinical applicability.

## Innovations and integrative approaches for precision oncology

Recent innovations seek to move beyond single biomarkers by developing multivariate extensions of the methods described above (e.g., multivariate Cox models or random survival forests incorporating multiple biomarkers), as well as multimodal frameworks with greater clinical relevance. Unlike traditional single-biomarker approaches, these methods assess the combined effects of multiple variables, allowing a more realistic representation of cancer’s biological complexity. For example, integrating genomic, transcriptomic, and proteomic data within the same analytical framework enables stratification based on a broader view of tumour biology. Practical implementation may also require harmonisation of heterogeneous data sources and careful handling of missing modalities, particularly in multi-centre datasets. By explicitly modelling interactions between variables, multivariate approaches can identify patterns that may be missed when biomarkers are considered in isolation [58]. This is particularly important in cancers where multiple pathways contribute to progression.

Clinically, these models are valuable because they bring risk assessment closer to the multidimensional decision-making process that oncologists face in practice. Rather than relying on a single threshold, clinicians can use integrated risk scores that account for diverse

**Table 1:** Comparative Overview of Methods for Biomarker Stratification in Survival Analysis

Method Class	Method Type	Method Name	Key Strengths	Key Limitations	Clinical Applicability	Reference
Rule-Based	Arbitrary	Quantile partitioning	Extremely simple; transparent thresholds (median/tertiles/quartiles); no modelling assumptions	Dichotomising/quantiling continuous variables loses information and power; thresholds are arbitrary and dataset-dependent; ignores censoring explicitly	Occasionally useful for quick stratification or descriptive reporting, but generally discouraged for prognostic modelling due to loss of discrimination and calibration	[15]
Data-Driven	Optimal Cut Point	ROC curve + Youden’s index	Single, data-driven threshold optimising (sensitivity + specificity − 1); easy to communicate	Optimises diagnostic accuracy, not time-to-event risk; prone to optimism if not cross-validated; may be unstable across cohorts	Reasonable for binary outcomes or fixed-time horizons; less suitable for survival endpoints without adapting to censoring	[52]
		Minimum p-value approach	Searches across cut points to maximise association with outcome; simple to implement	Severe multiple testing/Type I error inflation unless corrected; thresholds overfit; dichotomisation criticised in prognostic research	Use only with proper correction and external validation; continuous-scale modelling usually preferred	[33]
		Maximally selected rank statistics (MSRS)	Accounts for censoring using rank-based tests; provides adjusted p-values for cut point selection	Still reduces continuous predictors; threshold may be sample-specific; interpretability can be misleading as a universal biological cut-off	Useful when a clinically mandated threshold is needed but should be corroborated in validation cohorts	[29]
		Maximally selected Chi-square statistics (MSCS)	Framework for selecting cut points with adjusted significance using chi-square statistics	Shares the drawbacks of dichotomisation and instability; requires correction for searching over cut points	Consider when categorical decision rules are unavoidable and proper adjustment is applied	[34]
	Statistical Modelling	Cox-PH with threshold search	Handles censoring; widely used; effects are interpretable as hazard ratios; threshold search can be embedded via splines or change-points	Proportional hazards assumption may be violated; naive threshold search risks overfitting without penalisation/correction	Strong baseline for prognostic modelling and risk stratification when PH holds; flexible with time-varying effects/splines	[24]
		Generalised additive models (GAM)	Captures non-linear relationships via smooth functions; retains interpretability of smooths; can be combined with survival models	Requires careful smoothing parameter selection; extrapolation is uncertain; more complex than linear effects	Useful when clinical effect is clearly non-linear and interpretability of shape matters (e.g., lab values with plateaus)	[42]
Machine Learning	Unsupervised	Principal component analysis	Reduces dimensionality; alleviates multicollinearity; fast and deterministic; aids visualisation	Linear method; components may be hard to interpret clinically; ignores outcome during construction	Pre-processing for high-dimensional biomarkers; supports downstream modelling rather than direct stratification	[44]
		K-means clustering	Simple, scalable clustering; works well for spherical, well-separated clusters	Requires the value of k to be specified; sensitive to scaling and initialisation; assumes Euclidean structure; ignores censoring/outcomes	Exploratory patient subgrouping; must be validated for prognostic relevance post-hoc	[46]
		Hierarchical clustering	Does not require pre-specifying number of clusters; dendrogram aids clinical interpretation	Computationally heavier; choice of linkage/distance strongly affects results; outcome-agnostic	Useful for discovering phenotypes in omics/ICU data, to be linked to outcomes subsequently	[56]
		Latent class analysis	Model-based clustering with probabilistic class membership; handles mixed data types; supports uncertainty quantification	Model selection (number of classes) is non-trivial; results can be sensitive to starting values and local optima	Deriving clinical subtypes with posterior probabilities that can inform risk; can be linked to survival in a second stage	[47]
		Network-based methods	Captures complex relationships (e.g., patient-patient or feature networks); flexible integration of multi-omics	Choice of network construction and community detection affects robustness; interpretability varies	Phenotyping and pathway-level stratification in systems medicine; requires careful validation for prognostic use	[57]
	Supervised	K-nearest neighbours	Non-parametric; simple; naturally captures local structure	Sensitive to scaling and value of k; poor performance in high dimensions; no inherent handling of censoring	Baseline comparator for classification/regression; survival variants exist but are rarely used clinically	[43]
		Support vector machine	Strong performance with kernels on complex boundaries; regularisation controls overfitting	Hyperparameter tuning required; limited probabilistic interpretability; survival extensions increase complexity	When decision boundaries are complex and sample size is moderate; consider calibration methods for clinical use	[51]
		Classification and regression trees	Interpretable decision rules; handles mixed data; can incorporate missing-value surrogates	Unstable to small data perturbations; tendency to overfit without pruning; limited to axis-aligned splits	Useful for transparent triage or risk grouping; best paired with pruning/validation or ensemble methods	[48]
		Random survival trees	Ensemble of survival trees; handles censoring; captures non-linearities and interactions; robust to overfitting compared with single trees	Reduced interpretability; requires tuning; variable importance can be biased without correction	Strong choice for heterogeneous cohorts with complex effects; good discriminative performance for time-to-event outcomes	[53]
		Optimal survival trees	Interpretable tree tailored to survival objectives; aims for globally optimal splits rather than greedy growth	Computationally heavier than CART; implementations less mature; still axis-aligned splits	When a single, interpretable survival tree is desired with improved accuracy over greedy trees	[49]
		Bayesian network	Probabilistic graphical models enable causal reasoning and uncertainty quantification; handles missing data naturally	Structure learning is NP-hard; results depend on priors/assumptions; survival modelling requires specialised nodes	Decision support under uncertainty and scenario analysis; useful where expert knowledge can inform structure	[55]
		Deep learning	Learns complex non-linear representations; state-of-the-art in high-dimensional data (images, omics); survival-specific architectures exist	Data-hungry; computationally intensive; limited interpretability; risk of domain shift; careful calibration required	Promising for multimodal and high-dimensional prognostics; deployment requires rigorous validation and interpretability checks	[54]

information sources, supporting more robust and personalised treatment recommendations. In breast cancer, for instance, integrated models combining genomic signatures such as Oncotype DX with clinicopathological features including tumour size and nodal status have been shown to improve prognostic accuracy and refine chemotherapy decisions [23]. Similarly, in lung cancer, multimodal frameworks that merge imaging-derived features with molecular profiles are beginning to stratify patients more effectively for immunotherapy [56]. However, translation to single-patient classifiers remains challenging, as many studies - including multimodal NSCLC models - report cohort-level performance rather than validated individual risk scores [59].

While omics technologies have expanded the biomarker landscape, clinical covariates remain essential. Patient age, sex, comorbidities, tumour stage, and prior treatment history all influence prognosis. Models that integrate molecular biomarkers with clinical and pathological variables consistently outperform those using molecular data alone [55, 57]. This integrated approach reflects the real-world setting in which treatment decisions are made. For example, a gene expression profile may indicate aggressive disease biology, but if a patient has competing comorbidities or is unlikely to tolerate intensive therapy, the optimal treatment strategy may differ. Incorporating both molecular and clinical factors into prognostic models therefore helps ensure biomarker-driven recommendations remain feasible and clinically relevant.

However, additional biomarkers do not always translate into improved decision-making. PD-L1 expression illustrates this context-dependency: it is required for first-line atezolizumab or pembrolizumab eligibility in cisplatin-ineligible advanced urothelial cancer, but is unnecessary in later-line settings where broader populations benefit [60, 61]. The incremental clinical value of each biomarker therefore requires careful evaluation, including comparison with cheaper, faster histopathological alternatives. From a translational perspective, this type of integration also helps bridge the gap between research and practice. Models that incorporate routinely available clinical variables alongside novel biomarkers are more likely to gain acceptance and be implemented within oncology workflows. A practical example is the integration of molecular signatures with TNM staging in colorectal cancer, which has improved risk stratification and informed adjuvant chemotherapy treatment decisions [62]. In haematological malignancies, combining cytogenetic abnormalities with clinical variables has similarly enhanced prognostic accuracy and treatment tailoring [63].

A recurring challenge in biomarker research is overfitting - when models capture noise rather than true biological signal. This risk is particularly high in high-dimensional datasets, such as those generated by sequencing or proteomics, where the number of candidate variables far exceeds the number of patients. Overfitted models often show excellent performance in discovery datasets but fail in validation cohorts [11]. To counteract this, newer approaches increasingly emphasise dimensionality reduction, feature selection, and careful cross-validation. Techniques such as synthetic oversampling - which generates synthetic examples of rare event classes in imbalanced survival data - and ensemble modelling have been used to mitigate class imbalance and improve generalisability [39]. Importantly, these are not merely statistical refinements: they can directly shape clinical impact. Models that are robust to overfitting are more reliable in identifying which patients may benefit from specific therapies, reducing the risks of false reassurance or unnecessary treatment. Sparsity-promoting methods such as Stabl further support reliability by identifying minimal biomarker sets that generalise across cohorts [64].

Equally important is reproducibility, which remains a major barrier to translation. Internal validation strategies such as cross-validation and bootstrap resampling are widely used to assess stability in cut point selection or model performance. These methods generate repeated subsamples of the original dataset, enabling confidence interval estimation and reducing the likelihood that results reflect chance variation [65]. Even with such approaches, selected thresholds may remain unstable under resampling, reinforcing the need for validation across independent cohorts. External validation across independent cohorts remains the gold standard, providing reassurance that a risk score or stratification approach can be applied in routine practice without unexpected biases [40]. From a clinical perspective, this step ensures that a biomarker or model is not simply a statistical artefact, but a tool that can reliably inform patient care. Frameworks for model evaluation and reporting (e.g. calibration assessment and structured reporting guidelines such as TRIPOD) further support reproducibility and clinical translation [66].

Unfortunately, external validation is often neglected. Many candidate biomarkers are not tested beyond a single cohort, contributing to inconsistent findings and the high attrition rate seen during translation. Gene expression-based signatures for diffuse large B-cell lymphoma, for example, initially appeared promising but failed in broader validation due to overfitting and limited reproducibility [32]. The REMoDL-B trial offers a more nuanced case: gene expression profiling did not predict bortezomib clinical benefit when combined with chemoimmunotherapy, yet post-hoc analyses revealed prognostic value within molecular subtypes. This highlights how apparent validation failures can still reveal clinically relevant signals, dependent on subgroup context [67]. Without rigorous validation, even sophisticated methodologies risk producing results that are statistically compelling but clinically unreliable - and this helps to explain why many candidate biomarkers fail to influence clinical decision-making [11, 68].

Moving forward, embedding validation within biomarker research from the outset, rather than treating it as an afterthought, will be essential. Improving reproducibility will require transparent reporting, data sharing, and the adoption of standardised analytical pipelines. Clinicians need to trust that a biomarker identified in one population or hospital will perform similarly in another. Without this assurance, translation into routine care will remain limited. Large-scale consortia and data-sharing initiatives provide a path forward by enabling external testing across diverse datasets [69]. For patients, this ultimately means that predictive tools used in decision-making are not only innovative, but also dependable. A notable example is the MammaPrint assay in breast cancer, which achieved clinical uptake only after prospective external validation in large multicentre trials demonstrating reproducibility across diverse patient populations [9]. Such examples underscore the need for rigorous pipelines and cross-cohort testing prior to clinical adoption.

Overall, emerging innovations are reshaping biomarker stratification by shifting away from single thresholds towards integrated, multivariate frameworks that combine molecular and clinical data. By addressing overfitting, strengthening reproducibility, and prioritising validation, these approaches are increasingly designed with clinical translation in mind. Their ultimate goal is not methodological sophistication alone, but improved decision-making that delivers more precise and effective cancer care.

## Conclusions and future directions

Biomarkers remain central to tailoring treatment decisions and improving outcomes, but progress has been constrained by over-reliance on arbitrary cut points, inconsistent methodology, and limited validation. Data-driven and machine learning approaches now offer a richer toolkit for addressing continuous biomarkers, cohort heterogeneity, and high-dimensional data. In particular, multivariable and multimodal models demonstrate how combining molecular measurements with clinical covariates can yield more accurate and clinically meaningful predictions.

At the same time, important limitations remain. Many methods that perform well in research settings falter when applied to independent cohorts or real-world clinical practice. Overfitting, poor reproducibility, and insufficient external validation continue to undermine confidence in candidate biomarkers. For patients, this means that discoveries made through advanced computation or experimental profiling too often fail to translate into meaningful clinical benefit.

**Fig. 2:**
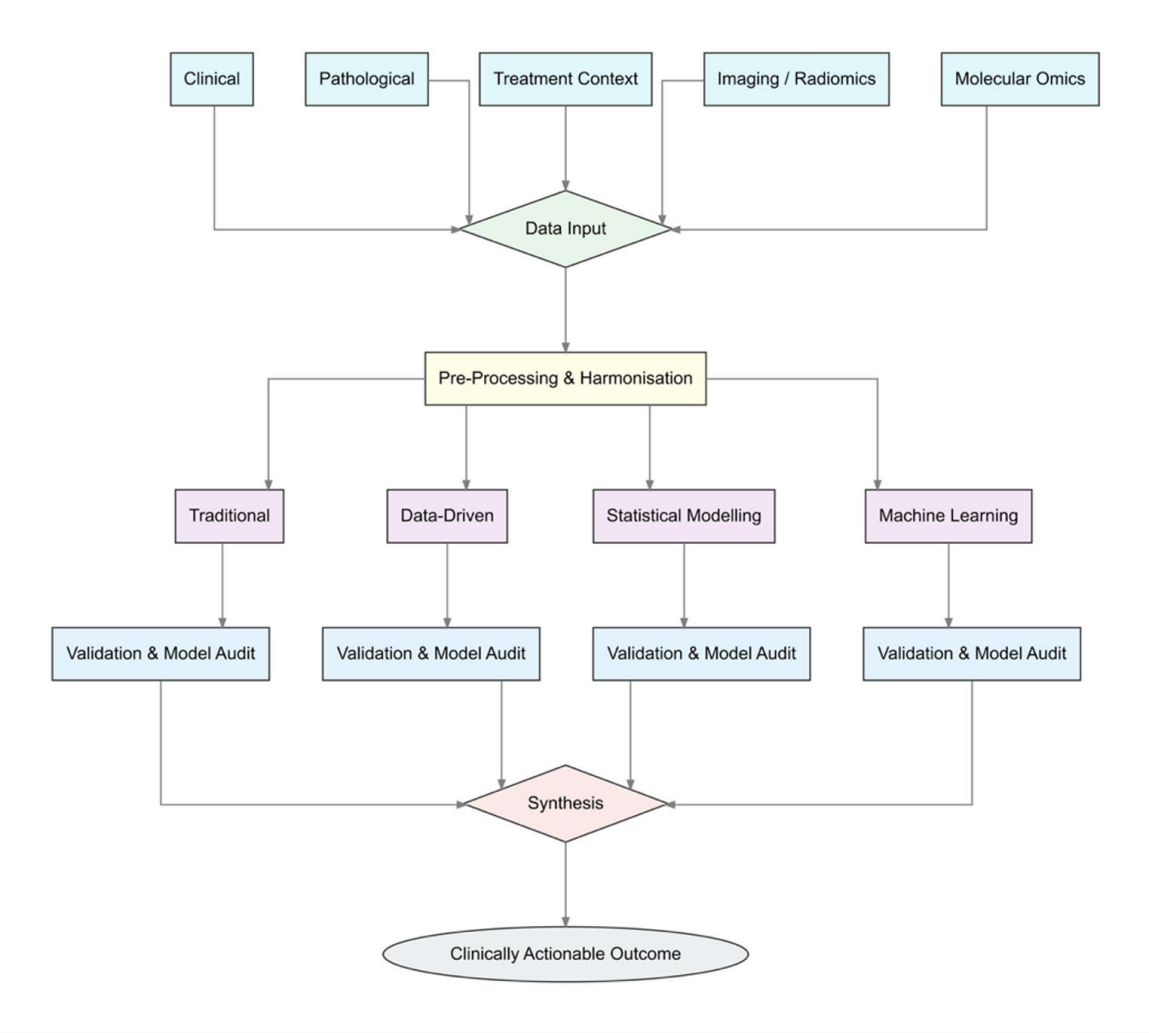
A Contemporary Framework for Biomarker Stratification in Precision Oncology. Here we present an integrative pipeline for translating biomarker-driven survival analysis into clinical practice. At the input level, a heterogeneous patient cohort contributes distinct but parallel data streams (teal rectangles), including clinical and pathological variables (e.g., age, stage, comorbidities, prior therapy), treatment context, imaging and radiomics features, and molecular omics (genomic, transcriptomic, proteomic). These can be used individually or in combination and are subject to pre-processing and harmonisation (yellow rectangle), encompassing quality control, missing-data handling, batch correction, class imbalance adjustment, and feature selection or dimensionality reduction. Downstream, four methodological families are illustrated: (i) traditional benchmarks, (ii) data-driven cut point identification, (iii) statistical modelling approaches, and (iv) machine learning, including unsupervised subgroup discovery and supervised predictive modelling (violet rectangles). Each approach is subject to validation and model audit through internal resampling (bootstrap, k-fold cross-validation), external multi-centre or temporal testing, assumption checks, calibration, discrimination, and decision-curve analysis (blue rectangles). Clinically actionable outputs include risk groups with confidence intervals, interpretable decision rules, integrated risk scores, and fairness/bias reporting, with attention to interpretability and integration into oncology workflows. Iterative feedback loops between outcomes and earlier steps (not shown on diagram) support continuous refinement and re-validation as patient populations and treatment strategies evolve, ensuring alignment with real-world practice

Future progress will require building on methodological advances while keeping clinical translation as the primary focus; prioritising frameworks that integrate multiple data types - combining molecular, clinical, and demographic variables - to provide a more holistic view of patient risk and treatment response. These principles are summarised in Fig. 2. This conceptual framework links heterogeneous patient data, analytical approaches, validation strategies, and clinically actionable outputs within a continuous learning loop.

Crucially, such models must emphasise interpretability and strike a careful balance between predictive power and clarity, ensuring outputs can be understood and trusted by clinicians making real-world decisions. Reproducibility should be addressed early through transparent reporting, data sharing, and multicentre collaboration, helping ensure findings generalise across populations and healthcare systems. Validation must also be treated as a standard requirement rather than an optional step, with external testing embedded into development pipelines before biomarkers are proposed for clinical use.

Rather than advocating for a single new methodology, the field would benefit from flexible frameworks that allow statistical, machine learning, and hybrid approaches to be compared, benchmarked, and adapted to specific clinical contexts. The emphasis should remain on methods that improve patient stratification in clinically actionable ways, rather than on computational sophistication alone. Ultimately, advances in biomarker stratification are not merely technical milestones: they are opportunities to refine cancer care. By ensuring methodological innovation remains closely tied to validation, reproducibility and interpretability, the next generation of biomarker-driven survival models can bring us closer to the central promise of precision oncology - delivering the right treatment to the right patient at the right time.

## Data Availability

No datasets were generated or analysed during the current study.
